# Centrally Acting ACE Inhibitor Use and Physical Performance in Older Adults

**DOI:** 10.14283/jfa.2023.10

**Published:** 2023

**Authors:** C.J. George, C.B. Hall, E.F. Weiss, J. Verghese, E. Neptune, P. Abadir

**Affiliations:** 1. Montefiore Medical Center, Division of Geriatrics, Albert Einstein College of Medicine, Bronx, New York, USA; 2. Department of Epidemiology and Population Health Albert Einstein College of Medicine Bronx New York USA; 3. Department of Neurology, Montefiore Medical Center and Albert Einstein College of Medicine, Bronx, NY, USA; 4. Division of Cognitive & Motor Aging and Geriatrics, Albert Einstein College of Medicine, Bronx, New York, USA; 5. Division of Pulmonary and Critical Care Medicine, John Hopkins University School of Medicine, Baltimore, MD, USA; 6. Division of Geriatric Medicine and Gerontology, Johns Hopkins University School of Medicine, Baltimore, MD, USA.

**Keywords:** Angiotensin-converting enzyme inhibitors, aging, gait, grip strength, peak flow

## Abstract

**BACKGROUND:**

There is conflicting evidence regarding the role of angiotensin-converting enzyme inhibitors and physical function. While some studies show improvements in muscle strength and physical function, others show no significant difference or decreased performance. This ambiguity could be due to differential effects of angiotensin-converting enzyme inhibitor subtypes which can be categorized as centrally or peripherally-acting based upon their ability to cross the blood-brain barrier.

**OBJECTIVE:**

The objective of this study is to compare physical performance measures among angiotensin-converting enzyme inhibitor subtype users.

**METHODS:**

Design: Cross-sectional Setting: Ambulatory Participants: Performed in 364 participants in the Health and Retirement Study cohort who were ≥ 65 years (median age (IQR) 74.00 (69-80) years. Measurements: Average difference in hand grip (kg), gait speed(m/s) and peak expiratory flow (L/min).

**RESULTS:**

Compared to participants on a peripherally-acting angiotensin-converting enzyme inhibitor (113 (31%)), those on a centrally-acting agent (251(69%)) had stronger grip strength 28.9 ±1.0 vs 26.3±1.0, p=.011 and higher peak expiratory flow rates 316.8±130.4 vs. 280.0±118.5, p= .011 in unadjusted analysis. After multiple adjustments the difference in PEF remained statistically significant (Estimate(CI) 26.5, 95% CI 2.24, 50.5, p = 0.032).

**CONCLUSION:**

Our results suggest that in older adults the use of centrally-acting angiotensin-converting enzyme inhibitors compared to a peripherally acting angiotensin-converting enzyme inhibitors was associated with better lung function in older individuals.

## Introduction

Older adults are particularly vulnerable to adverse health outcomes, including early mortality, functional decline, disability, and falls. The etiopathogenesis of age-related physical decline and the adverse outcomes associated with it are not well-defined, but has been linked to chronic inflammation, mitochondrial damage with bioenergetic failure, cellular senescence, and impaired autophagy in older adults (1).

The renin-angiotensin system (RAS), a central regulator of blood pressure and sodium balance, is involved (via increased Angiotensin II generation) in several molecular mechanisms that are linked to age-related loss of muscle mass and strength or sarcopenia, including chronic inflammation, oxidative stress damage, mitochondrial decline, and reduced blood flow to peripheral vascular beds (2). Angiotensin Converting Enzyme Inhibitors (ACEi) are a class of drugs that inhibit the production of angiotensin II. ACEi have established renal, cardiovascular, and blood pressure benefits and are in widespread clinical use (3). More recent evidence suggests that the RAS may play a role in the pathogenesis of pulmonary disease such as asthma and chronic obstructive pulmonary disease (4).

In contrast, the impact of angiotensin system blockade on physical function and lifespan remains unclear. Pharmacologic or genetic disruption of the angiotensin system in animal studies show reduced inflammation, enhanced mitochondrial energetics, improved muscle repair and physical performance, and led to a 25% increase in lifespan (5). However, in human studies, there is conflicting evidence in the literature regarding the impact of ACEi on physical function in older adults. While some studies show an improvement in muscle strength and physical function (6-9), others show no significant difference (10-12) or show an association with decreased physical performance (13-15).

In this study, we postulated that this ambiguity could be due to differential effects of ACEi subtypes. ACEi can be subcategorized as Centrally Acting Angiotensin Converting Enzyme Inhibitors (c-ACEi) or Peripherally acting Angiotensin Converting Enzyme Inhibitors (p-ACEi) based upon their ability to cross the blood-brain barrier. c-ACEi interact with the brain renin-angiotensin system(b-RAS), which is linked to both metabolic function and energy balance (16). Our hypothesis is that older adults on c-ACEi will have better measures of physical performance compared to those on p-ACEi due to the additional impact of c-ACEi on the b-RAS. We compared physical measures among c-ACEi and p-ACEi users in a nationally representative sample.

## Methods

### Participants

**Figure 1.**
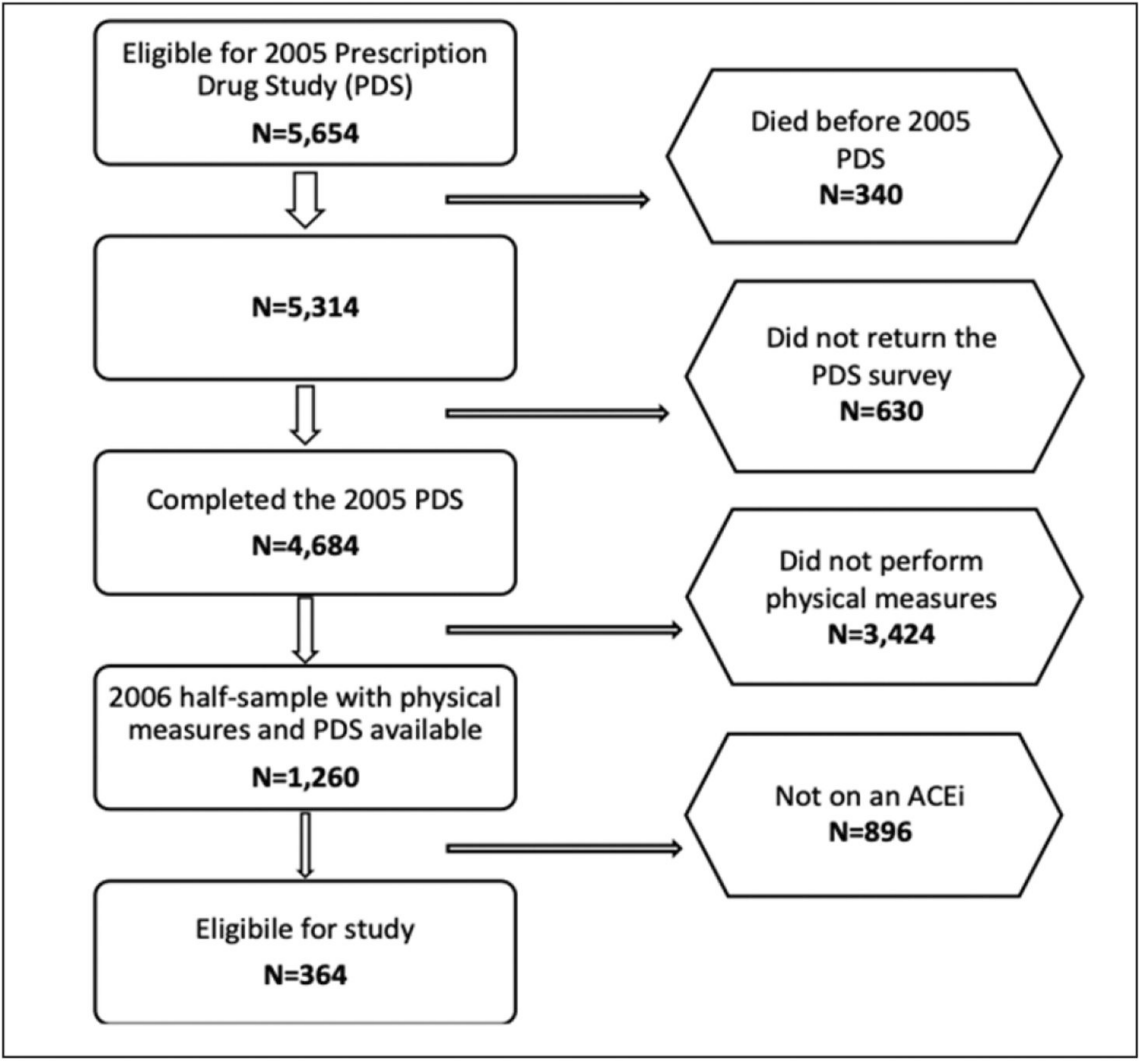
Participant Eligibility

We performed a cross-sectional study in community-dwelling older adults aged 65, and older enrolled in the Health and Retirement Study (HRS). The HRS is a longitudinal study sponsored by the National Institute of Aging and the Social Security Administration, which started in 1992 in the USA. It was established to provide a resource of data on changing health and economic circumstances associated with aging. The study design has been described previously (17). Briefly, participants born between 1931-1941 (age 51-61 at time of study initiation) were recruited and originally consented at the University of Michigan. Additional cohorts have been added since 1992 with a steady state recruitment design. Participants in the HRS complete biennial in home interviews with trained interviewers. The medication use information is available for a subset of participants who completed the 2004 wave and who were part of a Prescription Drug Study (PDS). Additionally, half of the 2004 participants had physical measures performed in 2006 as part of an Enhanced Face-to-Face (EFTF) interview. Participants from the 2004 assessment with both medications use and physical measures data were included in this study (Figure 1).

### Angiotensin Converting Enzyme Inhibitor Use

The HRS contains detailed medication use information through an off-cycle Prescription Drug Study (PDS) drawn from the 2004 wave to examine the impact of Medicare part D implementation. In HRS, 5,654 participants from the 2004 wave were randomly selected for the PDS mail survey (Figure 1). Inclusion criteria were age 65 years or older in 2006 when Medicare part D was implemented and not participating in another mail survey which was occurring at the same time. Three hundred and forty participants died prior to the PDS. Of the remaining 5,314 eligible participants, 4684 returned questionnaires or completed telephone interviews, for a response rate of 88.1%. The medication files contain drug names, dosage, cost, and questions about adherence to medication use.

The overall sample eligible for this study (those who completed the PDS and had 2006 physical measures) includes 1,260 participants, of which 364 were on an ACEi in 2005. ACEi use was categorized as c-ACEi or p-ACEi. c-ACEi include captopril, lisinopril, perindopril, fosinopril, trandolapril, zofenopril, and ramipril, while p-ACEi include enalapril, quinapril, benazepril, and moexipril.

### Physical Performance Measures

Physical measures were performed in 2006 as part of an Enhanced Face-to-Face (EFTF) interview on a random half sample of the eligible 2004 cohort. These measures were performed in the participants’ homes by trained study personnel. We identified a measure of upper extremity, lower extremity and truncal strength from available measures in HRS. Lung function, a reflection of truncal strength, was determined by peak flow or the peak expiratory flow (PEF) rate, measured 3 times, 30 seconds apart. Handgrip strength was attempted in both hands and measured in only one hand if the participant reported a condition that limited use of one hand such as surgery, swelling, inflammation or severe pain or injury within the last six months. Two measurements were taken and reported in kilograms. To assess lower extremity strength, participants were advised to walk at their normal pace on a 2.5-meter non-carpeted straight path. The time was recorded twice. The walking time was converted to speed and reported as gait speed in meters per second (m/s). Participants were allowed to use a walking aid such as a cane. Physical measures and physical performance measures with more than one recorded measurement were reported as an average of the available values.

### Other Covariates

Demographic characteristics, medical conditions, the presence of polypharmacy, high-risk medication use and antihypertensive medication use from HRS are included in this study. Information about medication use is obtained from the prescription drug survey as described above. Polypharmacy is defined as the use of 5 or more regularly scheduled medications. High risk medication use includes medications that are associated with falls and/or cognitive impairment and included benzodiazepines, first generation antihistamines, opioid analgesics, alpha-blockers, muscle relaxants, proton pump inhibitors, selective serotonin reuptake inhibitor, nonsteroidal anti-inflammatory drug, urinary antispasmotics and medications used to treat insomnia. Educational level is measured as years of schooling. Medical conditions are reported by the participant who answered ‘yes’ or ‘no’ if their doctor ever told them that they had a stated condition. A modified version of the Mini-Mental Status Exam (MMSE) forms the Total Cognitive Score (18), which measures overall cognitive function, and ranges from 0-35 (higher better). The Total Cognitive Score includes tests of immediate and delayed recall, serial 7s, counting backwards, object naming, recall of both the date and both the president and vice president. Physical measures include blood pressure measured in mmHg, pulse or heart rate in beats per minute (bpm) measured 3 times, 45 seconds apart. Weight and height were recorded and reported as BMI measured in kilogram per meters squared (kg/m^2^). Waist circumference (centimeter) was measured at the level of the navel.

### Statistical Analysis

The objective of the analysis was to examine the differences in physical performance measures in participants on a c-ACEi compared to those on a p-ACEi. Participants’ baseline characteristics were compared using descriptive statistics; two-sample T-test for continuous variables and Chi Square test for categorical variables. Third, we reported the results of linear regression models of the physical performance measures among ACEi subtype users. Adjustments were made for differences noted in Table 1, or based upon biological plausibility. Model assumptions were examined and met. Significance was determined to be a P value of 0.05 or less. All analysis was performed using SPSS version 26, IBM.

## Results

### Baseline Characteristics

The prevalence of ACEi use was 29% in the 1260 participants who completed the PDS and had 2006 physical measures. Among the 364 ACEi users, 251 (69%)were on a c-ACEi and 113(31%) on a p-ACEi. Table 1 describes the clinical characteristics of those on c-ACEi compared to those on p-ACEi. Differences between the two groups were minor. In the overall sample, the median age (IQR) was 74.00 (69-80) years, and the age range was 65 to 104 years. The sample was 51% female and 83% white. The prevalence of hypertension was 92%, diabetes 35%, and congestive heart failure 21%. The most commonly used antihypertensive medications in decreasing order of frequency were beta-blockers 33%, calcium channel blockers 18%, loop diuretics 16%, and thiazide diuretics 15%. There were no statistically significant difference in the presence of polypharmacy or high-risk medication use between the two groups.

### Physical Performance Measures

Compared to participants on a p-ACEi, those on a c-ACEi had a stronger grip strength (28.9±9.6 vs 26.3±9.7kg; p=0.021) and a higher peak expiratory flow rate (316.8±130.4 vs. 280.0±118.5 L/min; p=0.001) in unadjusted analysis (Table 2). There were no significant differences in gait speed.

Grip strength, gait speed and PEF were examined using linear regression to compare c-ACEi users to p-ACEi users (Table 3). For grip strength and Gait speed, there were no statistically significant differences among ACEi subtype users after adjusting for age, gender, and educational level, and additional adjustments for CHF or BMI. The differences in PEF remained significant (Estimate (CI) 26.0, 95% CI 2.15, 49.8, p=0.033) after adjusting for age, gender, educational level and race/ethnicity. Further adjustment for CHF (Table 3, Model 2, resulted in statistically significant differences in PEF 23.2 (95% CI 0.978, 45.4, p = 0.041). Adjustment for BMI, Model 4, resulted in statistically significant differences in peak flow (Estimate (CI)26.5 (2.2, 50.5) p=.032.

### Sensitivity Analysis

As a sensitivity analysis, 46 participants with lung disease were excluded in order to determine if the presence of lung disease was responsible for the observed differences, and the differences in PEF remained significantly higher among c-ACEi users (c-ACEi N=213, PEF 327.6 ±131.0; p-ACEi N=97, PEF 296.3 ±113.9, P=0.034).

To eliminate the potential impact of other blood pressure medications commonly prescribed with ACEi for the treatment of hypertension, we then restricted our analysis to participants who are on ACEi monotherapy. Specifically, we excluded participants from both groups who were also on beta-blockers, thiazide diuretics, calcium channel blockers, angiotensin receptor blockers, or loop diuretics. We then compared physical performance measures in participants who were on c-ACEi vs. p-ACEi. The numbers were small and the results were not statistically significantly different between the two groups. Compared to participants on a p-ACEi, those on a c-ACEi had a stronger grip strength of 30.6 ±9.4 (N=93) vs 27.3 ±10.3 (N=45), P=0.075, faster gait speed of 0.758 ± .236 (N=95) vs .733 ± .27 (N=41), P=0.607, and higher PEF of 334.4 ±137.6 (n=96) vs. 299.4 ±121.5 (n=51), P=0.115. The directions of the differences noted were the same as when ACEi use was not examined as monotherapy; however, these measured differences did not reach statistical significance in this smaller sample.

## Discussion

The overall prevalence of ACEi use in this community-based U.S. sample was 29%, which is similar to what has been described elsewhere (19). Approximately two-thirds were on c-ACEi and one-third on p-ACEi, which represents the proportion of available agents in the U.S and is not based upon clinical characteristics or medical comorbidities. There were differences in grip strength among ACEi subtype users that did not remain after adjusting for age, gender and educational level. c-ACEi users had higher PEF after adjusting for age, gender, educational level, race/ethnicity, in addition to CHF and BMI. The relationship between c-ACEi use and PEF was an interesting and significant finding. PEF is the maximal rate of forced exhalation after a full inspiration. While it can depend on the participant’s motivation, PEF reflects respiratory muscle strength (intercostal and abdominal) and large airway flow. Voluntary respiratory effort is controlled by the cerebral cortex and can be modulated by involuntary centers in the brainstem or by chemoreceptor stimulus. Angiotensin Type-1 receptors (AT1R) and Angiotensin Type-2 receptors (AT2R) have been identified in the cortex, hippocampus, basal ganglia and brainstem (20), which allows for an explanation regarding the differential effects of ACEi subtypes on pulmonary function. Age associated dysregulation of the balance between AT1R and AT2R in the b-RAS can lead to excessive neuroinflammation, oxidative stress and vascular dysfunction (20). Differential receptor abundance in the central respiratory centers may underscore the distinct effects of ACEi described here, and requires further investigation. Peak flow is easy to administer in the outpatient setting and could provide insight into the determination of performance levels and targeted prescribing in older adults regardless of documented lung disease. Furthermore, PEF has been shown to be a strong independent predictor of 5 year mortality in community dwelling older adults (21). The relationship between the RAS and inflammatory pulmonary disease has been described recently in both animal and human models (4). A study comparing captopril, a c-ACEi, to placebo showed that captopril resulted in a reduction in airway resistance in basal conditions in rats (22). In another study, ACEi enalapril reduced peak work rate response to exercise training in patients with COPD and without heart disease or diabetes who were randomized to receive enalapril or placebo (23).

To our knowledge, there is little to no data on the differential effects of ACEi subtypes on measures of physical performance, and specifically lung performance in older adults. c-ACEi interact with the b-RAS, which is linked to learning, memory, and both metabolic function and energy balance (16). In addition to systemic effects, c-ACEi inhibit the conversion of angiotensin I to angiotensin II in the b-RAS. Angiotensin II is also a potent vasoconstrictor in the brain that acts on at least two receptors, AT1R and AT2R. Most of the function of angiotensin II is carried out by the AT1R, which promotes vasoconstriction, oxidative stress, neuroinflammation and vascular remodeling (2). The AT2R does the opposite. AT1R levels in the b-RAS are upregulated in aging (2), which results in alteration of the balance between proinflammation and protective effects of the system (20). ACEi activity is further complicated by the presence of both a systemic RAS and locally expressed RAS which has been found in a number of tissues including the lungs, heart, vasculature and kidney (24).

When examining physical performance measures in older adults, some studies have only compared centrally acting ACEi to placebo (8, 10-12, 25), and others made no distinction between c-ACEi and p-ACEi (6) when compared to other agents. To our knowledge, no other study has directly compared c-ACEi to p-ACEi and physical performance in older adults. Our hypothesis that older adults on c-ACEi will have better measures of physical performance compared to those on a p-ACEi is supported by the reported contribution of ACEi actions on the b-RAS to decrease neuroinflammation and oxidative stress and alter metabolic function and energy balance.

### Strengths and limitations

This study is the first to comprehensively examine the association between physical performance measures and use of c-ACEi versus p-ACEi. An additional strength is the use of community dwelling older adults from a large nationally representative sample, and standardized assessments. While we adjusted for several potential confounders in our analyses, residual or unmeasured confounding may still be present. A randomized controlled trial design would more appropriately address confounding and dispel doubts on causality. It is important to note that clinicians do not prescribe ACEi based upon their ability to cross the BBB or subtype classification of c-ACEi or p-ACEi. Rather, ACEi are prescribed based upon physician familiarity with a specific agent and insurance formularies. Therefore the presence of unmeasured confounders when c-ACEi are compared to p-ACEi should not be a strong contributor. Differences in PEF could be attributed to underlying lung disease, however exclusion of participants with known lung disease resulted in a significant difference between ACEi subtype users suggesting that if underlying lung disease contributes, it only partially contributes to the differences among ACEi users. The small sample size is a limiting factor in this study. The next step is to expand this examining in a larger sample with additional measures of lung function. The cross-sectional design limits establishment of causation, but lays the foundation for future research into the longitudinal associations between physical performance measures and ACEi subtype use.

## Conclusion

Our results suggest a relationship between use of c-ACEi, but not p-ACEi, and PEF in a nationally representative sample of participants. Further investigation is needed to determine the factors contributing to the differential effects of ACEi subtypes on this performance measure in older adults.

## Figures and Tables

**Table 1:** Baseline Characteristics of Participants on c-ACEi and p-ACEi N=364

	Total N=364	c-ACEi N=251(69)	p-ACEi N=113(31)	P value
Age, years (Median, IQR)	74.00 (69-80)	73(69 −79)	73 (69-80)	0.729
Gender (Female)	184 (50.5)	119 (47.4 )	65 (57.5)	0.089
Educational Level, years(Median, IQR)	12(11-14)	12 (11-14 )	12 (12-14)	0.754
Race/Ethnicity (n,%)				
White	302(83.0)	210(83.7)	92(81.4)	0.337
Black	48(13.2)	29(11.6)	19(16.8)	
Other	14(3.3)	12(0.5)	2(1.8)	
Hispanic (n,%)	22(6.2)	18(7.3)	4(3.7)	0.237
Comorbidities (n,%)				
Hypertension	335(92)	231(92)	104(92)	1.000
Diabetes	128(35.2)	90(35.9)	38(33.6)	0.723
Stroke	34(9.4)	25(10.1)	9(8)	0.567
Lung disease	46(12.6)	30(12)	16(14.2)	0.610
Congestive Eleart Failure	29(21.2)	23(21.1)	6(21.4)	1.000
Psychiatric, emotional, or nervous condition	62(17)	43(17.1)	19(16.8)	1.000
Fall within 2 years	122(33.7)	84(33.7)	38(33.6)	1.000
Arthritis	272(74.7)	190(75.7)	82(72.6)	0.518
Incontinence	84(23.1)	59(23.5)	25(22.1)	0.893
Heart Attack	16(11.5)	13(11.8)	3(10.3)	1.000
Memory related disease	4(1.1)	3(1.3)	1(0.9)	1.000
Total Cognitive Score* (m)	21.24 (5.2)	21.23 (5.2)	21.28 (5.3)	0.936
Physical Measures (Mean(SD))				
Systolic Blood Pressure†	133.76± 20.6	133.8±22.5	133.3±20.1	0.852
Diastolic Blood Pressure†	77.8 ±11.5	76.6.0±12.1	77.0±12.1	0.782
Pulse (bpm)‡	69.4±11.6	69.0±11.5	70.8±13.3	0.253
Body Mass Index (Kg/m^2^)§	28.8±5.6	29.1±5.8	29.1±6.4	0.971
Waist Circumference (Inches)∥	39.8±5.8	40.7±6.2	40.6±6.1	0.879
Medication Use (n,%)				
Polypharmacy (5 or more)	196(53.8)	139(55.4)	57(50.4)	0.427
High-Risk Medication	151(41.5)	107(42.6)	44(38.9)	0.566
ARB	9(2.5)	6(2.4)	3(2.7)	1.000
Loop Diuretic	59(16.2)	42(16.7)	17(15)	0.760
Thiazide Diuretic	57(15.7)	37(14.7)	20(17.7)	0.533
Potassium Sparing	7(1.9)	5(2.0)	2(1,8)	1.000
Beta blocker	117(32.1)	86(34.3)	31(27.4)	0.226
Calcium Channel Blocker	67(18.4)	51(20.3)	16(14.2)	0.189

* Total Cognitive Score, N=247 in c-ACEi; 109 in p-ACEi.

† Blood pressure N= 242 in c-ACEi; 104 in p-ACEi.

‡ Pulse N=242 in c-ACEi; 103 in p-ACEi.

§ BMI N=230 in c-ACEi; p-ACEi 107.

∥ Waste Circumference N=240 in c-ACEi; 109 in p-ACEi.

**Table 2:** Unadjusted Comparison of Performance Measures for c-ACEi and p-ACEi Users

Physical Performance Measure	c-ACEi Mean ± SD	p-ACEi Mean ±SD	Mean Difference, 95% CI, P-value
Grip Strength (Kg)*	28.9±9.6	26.3 ±9.7	2.67 (0.40 - 4.93) 0.021
Gait Speed (m/s)†	0.75±0.3	0.73±0.3	0.02 (−0.04 - 0.08) 0.590
Peak Expiratory Flow (L/min)‡	316.8±130.4	280.0±118.5	36.00 (8.28 - 63.68) 0.011

* Grip Strength N=235 in c-ACEi; 101 in p-ACEi.

† Gait Speed N= 226 in c-ACEi; 96 in p-ACEi.

‡ Peak Expiratory Flow N=239 in c-ACEi;111 in p-ACEi

**Table 3:** Adjusted Comparison of Performance Measures using Linear Regression

	*Model 1 Estimate (95% CI); P value	†Model 2 Estimate (95% CI); P value	‡Model 3 Estimate (95% CI); P value
Grip strength	1.3 (−0.0283, 2.92) 0.107	1.5 (−0.132, 6.14) 0.060	1.5 (−0.150, 3.088) 0.075
Gait Speed	0.024 (−0.032, 0.080) 0.396	0.081 (−0.033, 0.195) 0.163	0.026 (−0.030, 0.081) 0.360
PEF	26.0 (2.15, 49.8) 0.033	23.2 (0.978, 45.4) 0.041	26.49 (2.24, 50.5) 0.032

* Model 1: Age, gender, educational level, Race/Ethnicity

† Model 2: Age, gender, educational level, Race/Ethnicity, CHF

‡ Model 3: Age, gender, educational level, Race/Ethnicity, BMI
